# Transvaginal ultrasound and magnetic resonance imaging in detecting rectosigmoid deep infiltrating endometriosis: a comparative meta-analysis

**DOI:** 10.3389/fmed.2025.1552185

**Published:** 2025-03-17

**Authors:** Ziwei Xu, Yisheng Li, Yingying Wang, Yiting Wan, Jing Chen

**Affiliations:** ^1^Department of Gynecology, Shanghai Municipal Hospital of Traditional Chinese Medicine, Shanghai University of Traditional Chinese Medicine, Shanghai, China; ^2^Shanghai University of Traditional Chinese Medicine, Shanghai, China; ^3^Changfeng Community Health Service Center, Shanghai, China

**Keywords:** ultrasonography, magnetic resonance imaging, rectosigmoid, endometriosis, meta-analysis

## Abstract

**Objective:**

This meta-analysis aimed to assess the diagnostic efficacy of transvaginal ultrasound (TVS) and magnetic resonance imaging (MRI) for the detection of rectosigmoid deep infiltrating endometriosis (DIE).

**Methods:**

A thorough systematic review was performed by searching the PubMed and Embase databases for studies evaluating the diagnostic performance of TVS and MRI in rectosigmoid DIE, up until August 12, 2024. The DerSimonian and Laird approach was utilized to calculate sensitivity and specificity, with the Freeman-Tukey double arcsine transformation employed for data analysis. The quality of the included studies was evaluated using the Quality Assessment of Diagnostic Accuracy Studies-2 (QUADAS-2) tool.

**Results:**

The meta-analysis encompassed 10 studies involving 1,604 patients. The findings revealed that TVS had an overall sensitivity of 0.85 (95% CI: 0.76–0.92) and specificity of 0.92 (95% CI: 0.85–0.98), while MRI demonstrated a sensitivity of 0.83 (95% CI: 0.73–0.92) and specificity of 0.95 (95% CI: 0.90–0.99). Statistical analysis indicated no significant differences in sensitivity (*p* = 0.86) or specificity (*p* = 0.50) between the two imaging techniques. Additionally, the funnel plot asymmetry test did not reveal significant publication bias for any outcomes (Egger’s test: all *p* > 0.05).

**Conclusion:**

The meta-analysis reveals nearly equivalent diagnostic performance of TVS and MRI in detecting rectosigmoid DIE, with no statistical differences in sensitivity and specificity. However, high heterogeneity among studies highlights the need for further prospective research.

**Systematic review registration:**

The protocol for this meta-analysis has been registered with the International Prospective Register of Systematic Reviews (PROSPERO) under the ID: CRD42024559141, https://www.crd.york.ac.uk/PROSPERO/view/CRD42024559141.

## 1 Introduction

Rectosigmoid deep infiltrating endometriosis (DIE) is a severe form of endometriosis that involves the bowel, specifically affecting the rectosigmoid colon (1). This condition is characterized by the infiltration of endometrial-like tissue into the bowel wall. It leads to significant morbidity, including chronic pelvic pain, dyschezia, and bowel obstruction (2). The prevalence of rectosigmoid DIE among women with endometriosis is reported to be approximately 5–12%, making it a relatively common manifestation of this disease (3). Therefore, early and accurate diagnosis is vital in guiding appropriate management. Timely identification enables clinicians to determine whether medical therapy or surgical intervention is required, which can significantly enhance patient outcomes and quality of life (4).

Traditionally, the diagnosis of rectosigmoid DIE has involved a combination of clinical evaluation and various imaging techniques, including computed tomography, biopsy, and rectal endoscopic sonography. However, each of these methods has significant limitations in the context of DIE. Computed tomography, while useful for general pelvic imaging, lacks the soft-tissue contrast necessary to accurately delineate endometriotic lesions, particularly those involving the bowel. This limitation reduces its sensitivity and specificity for diagnosing DIE (5). Biopsy, though definitive, is invasive and often difficult to perform on deep lesions, which can lead to sampling errors and a higher risk of complications (6). Rectal endoscopic sonography may not effectively differentiate between DIE and other forms of bowel pathology, such as malignancies or inflammatory diseases, limiting its diagnostic performance (7). These limitations highlighted the need for more effective, less invasive diagnostic tools for the early detection and management of rectosigmoid DIE.

In recent years, the comparison between magnetic resonance imaging (MRI) and transvaginal ultrasound (TVS) for the diagnostic performance of DIE has become an emerging area of researches. MRI is renowned for its superior soft-tissue contrast and multiplanar imaging capabilities, which allow detailed assessment of the pelvic anatomy and the extent of disease (8). TVS, on the other hand, is a readily available, cost-effective, and patient-friendly tool that can provide real-time imaging with high resolution (9). Despite their widespread use, there is ongoing debate regarding the diagnostic performance of MRI versus TVS in detecting rectosigmoid DIE. In addition to their diagnostic capabilities, both TVS and MRI play important roles in the follow-up examinations of patients with rectosigmoid deep infiltrating endometriosis. TVS can be utilized for regular monitoring due to its accessibility and ability to provide immediate feedback on changes in the condition (10). MRI, with its detailed imaging, is particularly useful for assessing the extent of disease progression and planning further management strategies (11). The literature presents different findings, with some studies favoring MRI for its comprehensive imaging capabilities, while others suggest that TVS may offer comparable diagnostic performance, particularly when performed by experienced operators (8, 9, 12, 13).

The purpose of this meta-analysis is to systematically compare the diagnostic performance of MRI and TVS in detecting rectosigmoid DIE, aiming to provide evidence-based recommendations for clinical practice.

## 2 Methods

This meta-analysis adhered rigorously to the Preferred Reporting Items for Systematic Reviews and Meta-analyses: Diagnostic Test Accuracy (PRISMA-DTA) guidelines (14), ensuring comprehensive and transparent methodological reporting of diagnostic research. The protocol for this meta-analysis has been registered with the International Prospective Register of Systematic Reviews (PROSPERO) under the ID CRD42024559141.

### 2.1 Search strategy

A thorough search of the PubMed and Embase databases was performed to identify relevant studies up from August 1994 to August 12, 2024. The search was based on specific key terms, including “endometriosis,” “ultrasonography,” and “magnetic resonance imaging.” The detailed search strategy can be found in Supplementary Table 1. In addition to the database search, the reference lists of the identified articles were manually reviewed to identify any additional studies that may not have been captured in the initial search. This combined approach was employed to ensure the inclusion of all potentially eligible studies.

### 2.2 Inclusion and exclusion criteria

Studies were eligible to be included: Population (P): Patients suspected of rectosigmoid DIE; Intervention (I): TVS; Comparison (C): MRI; Outcome (O): Sensitivity and specificity; Study design (S): Retrospective or prospective studies.

Studies were excluded from this analysis based on several criteria to ensure the relevance and quality of the included data. First, articles were excluded if they lacked full texts, had irrelevant titles or abstracts, or were identified as duplicates. Publications such as case reports, letters, reviews, meta-analyses, non-English articles, and editorial comments were also excluded. In addition, studies that did not provide the necessary data for calculating key diagnostic outcomes, sensitivity and specificity, were excluded. Studies that did not involve direct head-to-head comparisons of the imaging modalities were also excluded. For studies with potentially overlapping patient populations, only the most recent study was included to ensure the inclusion of the most up-to-date data.

### 2.3 Quality assessment

We employed the QUADAS-2 framework to systematically evaluate the methodological quality of diagnostic accuracy studies. The assessment focused on four critical domains: patient selection, indicator testing, reference criteria and process, and timing (15). To ensure methodological rigor, two independent researchers conducted comprehensive quality evaluations, with any interpretative discrepancies resolved through collaborative deliberation or third-party adjudication.

The assessment protocol involved customizing domain-specific evaluation criteria aligned with QUADAS-2 guidelines. These tailored questions were strategically designed to critically examine potential biases and assess the overall applicability of primary diagnostic studies within the systematic review. Bias risk was stratified into three hierarchical categories: “high risk,” “low risk,” and “unclear risk.”

### 2.4 Data extraction

We extracted the following data from the selected studies: the author, publication year, country of the study, study design (prospective or retrospective), reference standard (surgery, histopathology), number of observers, number of patients, mean or median age of patients, cases with rectosigmoid DIE, imaging method of MRI technique and imaging method of TVS technique.

To ensure methodological integrity, two independent researchers conducted simultaneous data extraction, implementing a robust verification mechanism. A collaborative consensus-building approach was employed to resolve potential interpretative discrepancies.

### 2.5 Outcome measures

In this meta-analysis, the primary focus was on evaluating the diagnostic performance of TVS and MRI in the detection of rectosigmoid DIE. The study assessed the sensitivity and specificity of both imaging technique. Sensitivity is defined as the proportion of true positives (TP) identified by the imaging method, relative to the sum of TP and false negatives (FN). This measure reflects the ability of the imaging technique to correctly identify cases of rectosigmoid DIE. On the other hand, specificity refers to the proportion of true negatives (TN) detected by the imaging technology, in relation to the total number of TN and false positives (FP). Specificity quantifies the performance of the imaging method in correctly ruling out individuals without rectosigmoid DIE.

### 2.6 Statistical analysis

Meta-analytical techniques were applied to quantify diagnostic performance, employing the DerSimonian and Laird statistical approach for estimating pooled specificity and sensitivity. The Freeman-Tukey double arcsine transformation was implemented to normalize the diagnostic performance metrics. Confidence intervals were computed utilizing the Jackson method, which provides robust interval estimation. Statistical heterogeneity was comprehensively assessed through Cochrane Q and I^2^ statistical measures (16). When significant inter-study heterogeneity was detected (defined by *p* < 0.10 or I^2^ > 50%), a leave-one-out sensitivity analysis was implemented through sequential article exclusion and subsequent reassessment of sensitivity and specificity. Meta-regression analysis was strategically employed to explore potential sources of heterogeneity.

Publication bias was rigorously evaluated using funnel plot visualization and Egger’s statistical test (17). All statistical analysis were conducted by using R software (version 4.4.0), and the quality assessment was conducted using Revman 5.3 software.

## 3 Results

### 3.1 Study selection

The initial search identified 1,488 potential articles, supplemented by 2 additional articles discovered through alternative references. Preliminary screening eliminated 293 duplicate articles. Subsequent application of predefined inclusion criteria further narrowed the selection, excluding 1,175 articles, resulting in 22 articles meeting initial screening requirements.

**Figure 1:**
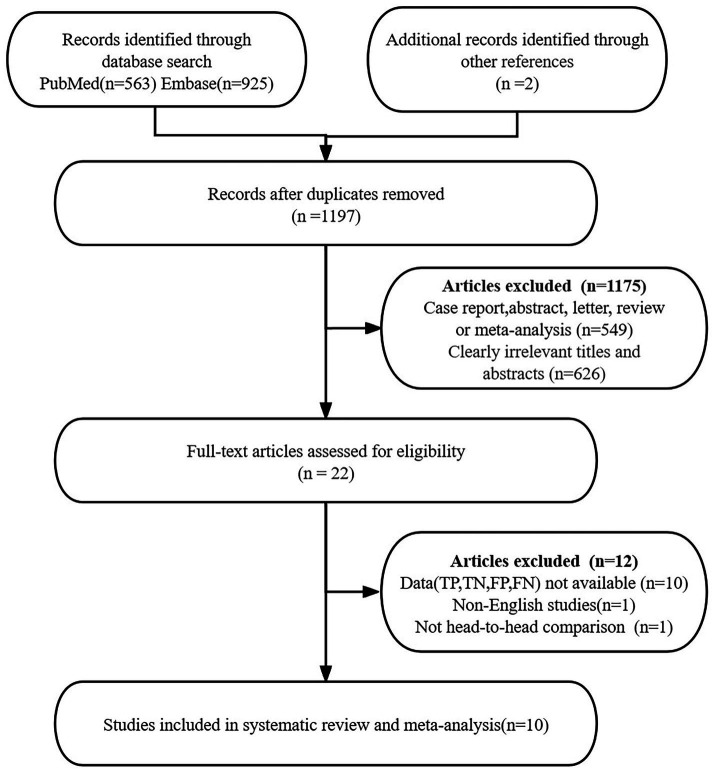
Flow chart of literature search and study selection.

A meticulous full-text review initiated a critical evaluation phase, leading to the exclusion of 12 additional articles based on specific methodological criteria. Exclusion rationales encompassed critical research limitations, including: incomplete diagnostic data sets (*n* = 10); non-English language publications (*n* = 1); not head-to-head comparison (*n* = 1). The final analytical cohort comprised 10 articles specifically focused on evaluating the diagnostic performance of TVS and MRI in rectosigmoid DIE detection (18–27). The selection process adhered to PRISMA guidelines, ensuring a structured, transparent, and reproducible approach to literature curation. A detailed flow diagram (Figure 1) comprehensively illustrated the article selection process.

### 3.2 Study description and quality assessment

This meta-analysis encompassed a diverse cohort of 10 eligible studies, involving 1,604 patients (ranging from 33 to 555, with a median of 98). Among these, 5 (50%) were retrospective studies, and 5 (50%) were prospective. Regarding number of patients, 5 (50%) studies analyzed more than 100 patients and 5 (50%) studies analyzed fewer than 100 patients. For the reference standard, these studies all used surgery and histopathology as the diagnostic gold standard. Detailed patient characteristics and study-specific information, and technical aspect were comprehensively documented in Tables 1, 2.

**Table 1:** Characteristics of studies and patients in all included studies.

Author	Country	Study design	Reference standard	No. of observers	No. of patients	Mean/Median age	Cases with rectosigmoid DIE	MRI technique	TVS technique
Roditis et al. (20)	France	Retro	Surgery and histopathology	2	178	Mean(range): 32.8 (19–49)	61	1.5 T or 3 T	TVUS
Abrao et al. (27)	Brazil	Retro	Surgery and histopathology	2	104	Mean ± SD: 33.8 ± 6.1	54	1.5 T	TVUS
Guerriero et al. (30)	Italy	Pro	Surgery and histopathology	2	159	Mean ± SD: 33 ± 7	75	1.5 T	3DTVUS
Saba et al. (19)	Italy	Pro	Surgery and histopathology	3	59	Mean(range):33 (21–44)	30	1.5 T	TVUS
Maggiore et al. (21)	Italy	Pro	Surgery and histopathology	2	286	Mean ± SD: 31.9 ± 4.8	151	1.5 T	RWC-TVS
Gutiérrez et al. (22)	Spain	Retro	Surgery and histopathology	NA	48	Mean ± SD: 34 ± 6	32	1.5 T	TVUS
Bazot et al. (25)	France	Retro	Surgery and histopathology	4	92	Median (range): 31.8 (20–51)	63	1.5 T	TVUS
Grasso et al. (24)	Italy	Pro	Surgery and histopathology	2	33	Mean (range): 35 (22–53)	4	1.5 T	3DTVUS
Alborzi et al. (26)	Iran	Retro	Surgery and histopathology	2	555	Mean (range): 34.13 (33.20–35.07)	534	1.5 T	TVUS
Vimercati et al. (18)	Italy	Pro	Surgery and histopathology	3	90	Mean ± SD: 34.3 ± 6.0	16	1.5 T	TVUS

TVUS, Transvaginal ultrasonography; Pro, prospective; Retro, retrospective; RWC, rectal water contrast; NA, not available.

**Table 2:** Technical aspects of included studies.

Author	TVS				MRI			
	TP	FP	FN	TN	TP	FP	FN	TN
Roditis et al. (20)	51	8	10	109	53	5	8	112
Abrao et al. (27)	53	0	1	50	45	1	9	49
Guerriero et al. (30)	65	16	10	68	66	14	9	70
Saba et al. (19)	22	4	8	25	22	3	8	26
Maggiore et al. (21)	140	4	11	131	144	3	7	132
Gutiérrez et al. (22)	26	6	6	10	22	2	10	14
Bazot et al. (25)	59	NA	4	NA	55	NA	8	NA
Grasso et al. (24)	1	0	3	29	3	0	1	29
Alborzi et al. (26)	371	2	163	19	274	4	260	17
Vimercati et al. (18)	12	6	4	68	16	0	0	74

TP, true positive; TN, true negative; FP, false positive; FN, false positive; NA, not available.

The comprehensive methodological evaluation utilized the QUADAS-2 tool to systematically assess research quality and potential bias across the included studies. Detailed bias risk characteristics were comprehensively documented in Table 3. In the aspect of index testing, 8 (80%) studies were judged as “unclear” because it was unclear whether a pre-determined cut-off value was used. With regards to the aspect of flow and timing, 1 (10%) study was judged as “high risk” because the time interval between some diagnostic tests and the gold standard was more than 3 months. Overall, the qualities of the included studies were deemed acceptable.

**Table 3:** QUADAS-2 quality evaluation form.

Author	Risk of bias				Applicability concerns		
	Patient selection	Index test	Reference standard	Flow and timing	Patient selection	Index test	Reference standard
Roditis et al. (20)	L	U	L	H	L	L	L
Abrao et al. (27)	L	L	L	L	L	L	L
Guerriero et al. (30)	L	U	L	L	L	L	L
Saba et al. (19)	L	U	L	L	L	L	L
Maggiore et al. (21)	L	U	L	L	L	L	L
Gutiérrez et al. (22)	L	U	L	L	L	L	L
Bazot et al. (25)	L	U	L	L	L	L	L
Grasso et al. (24)	L	U	L	L	L	L	L
Alborzi et al. (26)	L	U	L	U	L	L	L
Vimercati et al. (18)	L	L	L	L	L	L	L

L, low risk; U, unclear; H, high risk.

### 3.3 Comparing the sensitivity of TVS and MRI in diagnosing rectosigmoid DIE

**Figure 2:**
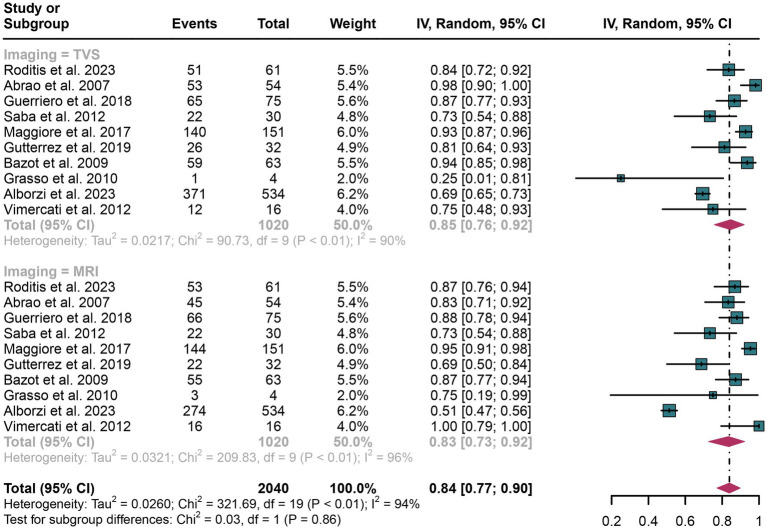
Forest plot showing the analysis for the sensitivity of TVS and MRI in diagnosing rectosigmoid DIE. The squares in the figure represent the estimated values of each individual study, the size of the squares indicates the relative weight of each study in this meta-analysis, the horizontal lines represent the corresponding 95% confidence intervals, and the diamonds represent the aggregated combined sensitivity estimates.

The pooled sensitivity of TVS was calculated to be 0.85 (95% CI: 0.76–0.92), while for MRI, it was 0.83 (95% CI: 0.73–0.92) (Figure 2). There was no statistically significant difference between the sensitivities of TVS and MRI (*p* = 0.86) (Figure 2).

The I^2^ statistic for TVS sensitivity was 90%. A leave-one-out sensitivity analysis did not identify the potential sources of heterogeneity (Supplementary Figure 1). Moreover, a meta-regression analysis was performed to explore possible factors contributing to heterogeneity, with results indicating that none of the three covariates—study design, patient number, or geographical region—significantly affected TVS sensitivity (Table 4).

**Table 4:** Meta-regression analysis of factors affecting the sensitivity and specificity of transvaginal ultrasonography.

Covariate	Studies, n	Sensitivity (95%CI)	*p*-value	Studies, n	Specificity (95%CI)	*P*-value
Number of patients included			0.29			0.44
>100	5	0.87(0.76;0.95)		5	0.94(0.86;0.99)	
≤100	5	0.79(0.62;0.93)		4	0.89(0.71;1.00)	
Region			0.12			0.77
Asian	1	0.69(0.66;0.75)		1	0.90(0.69;0.98)	
Non-Asian	9	0.87(0.79;0.94)		8	0.93(0.84;0.98)	
Study design			0.43			0.83
Retrospective	5	0.87(0.74;0.95)		4	0.91(0.73;1.00)	
Prospective	5	0.81(0.65;0.94)		5	0.93(0.84;0.98)	

For MRI sensitivity, the I^2^ value was 96%, and the leave-one-out sensitivity analysis also revealed no clear source of heterogeneity (Supplementary Figure 2). However, meta-regression suggested that the geographical region (*p* < 0.01) could be a significant factor contributing to the heterogeneity in MRI sensitivity (Table 5).

**Table 5:** Meta-regression analysis of factors affecting the sensitivity and specificity of magnetic resonance imaging.

Covariate	Studies, n	Sensitivity (95%CI)	*P*-value	Studies, n	Specificity (95%CI)	*P*-value
Number of patients included			0.98			0.45
>100	5	0.83(0.66;0.94)		5	0.93(0.86;0.98)	
≤100	5	0.84(0.69;0.96)		4	0.97(0.88;1.00)	
Region			<0.01			0.13
Asian	1	0.51(0.47;0.56)		1	0.81(0.58;0.93)	
Non-Asian	9	0.88(0.80;0.94)		8	0.96(0.91;0.99)	
Study design			0.23			0.47
Retrospective	5	0.76(0.61;0.89)		4	0.94(0.85;0.99)	
Prospective	5	0.91(0.79;0.99)		5	0.96(0.88;1.00)	

### 3.4 Comparing the specificity of TVS and MRI in diagnosing rectosigmoid DIE

**Figure 3:**
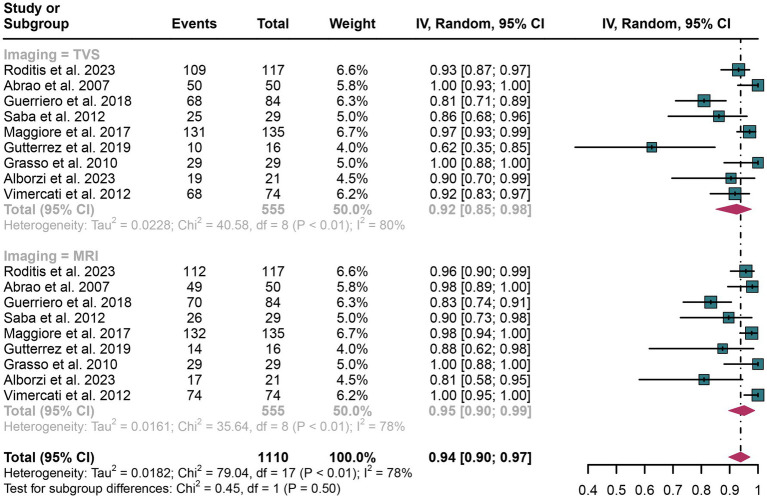
Forest plot showing the analysis for the specificity of TVS and MRI in diagnosing rectosigmoid DIE. The squares in the figure represent the estimated values of each individual study, the size of the squares indicates the relative weight of each study in this meta-analysis, the horizontal lines represent the corresponding 95% confidence intervals, and the diamonds represent the aggregated combined specificity estimates.

In the assessment of rectosigmoid DIE, TVS demonstrated an overall specificity of 0.92 (95% CI: 0.85–0.98), whereas MRI showed a pooled specificity of 0.95 (95% CI: 0.90–0.99) (Figure 3). The difference in specificity between TVS and MRI was not statistically significant (*p* = 0.50) (Figure 3).

For TVS, the I^2^ value for specificity was 80%. A leave-one-out sensitivity analysis did not pinpoint any potential sources of heterogeneity (Supplementary Figure 3). Additionally, a meta-regression analysis examining three covariates—study design, patient count, and geographical region—revealed that none had a significant impact on the specificity of TVS (Table 4).

In the case of MRI, the I^2^ value for specificity was 78%. The leave-one-out sensitivity analysis similarly did not identify any sources of heterogeneity (Supplementary Figure 4). Furthermore, meta-regression analysis indicated that none of the covariates significantly influenced the specificity of MRI (Table 5).

### 3.5 Publication bias

The funnel plot asymmetry test did not reveal significant publication bias for any outcomes (Egger’s test: all *p* > 0.05; Supplementary Figures 5–8).

## 4 Discussion

In 2014, Piessens et al. (13) found that TVS is the most commonly studied and frequently used imaging method for the preoperative diagnosis of DIE. TVS is preferred due to its accessibility, low cost, and non-invasive nature. However, in 2023, Rousset et al. (28) established that MRI remains the gold standard for imaging in patients with DIE, recommending the use of standardized MRI segmental structured reports to ensure performance. Despite these guidelines, recent studies have demonstrated inconsistent diagnostic performance between TVS and MRI for diagnosing rectosigmoid DIE. Gerges et al. suggested that TVS might offer slightly better sensitivity than MRI for detecting rectal or rectosigmoid involvement (29). Conversely, Guerriero et al. systematically evaluated the diagnostic performance of these imaging modalities in multiple anatomical regions, including the rectosigmoid region, uterosacral ligaments, and rectovaginal septum. They evaluated and compared diagnostic methods and concluded that TVS and MRI had similar diagnostic performance (30). Therefore, there remains a lack of systematic head-to-head comparisons between these two modalities, and the question of which diagnostic tool provides superior performance for rectosigmoid DIE remains unresolved.

This meta-analysis included 10 studies with a total of 1,604 patients, and the findings demonstrated that both TVS and MRI have similar diagnostic performance for detecting rectosigmoid DIE. The similar performance of TVS and MRI can be attributed to several factors. TVS, despite being a less invasive and more cost-effective technique, allows for high-resolution imaging of pelvic structures, making it highly effective in detecting endometriotic lesions, especially in the rectosigmoid region (31). TVS can screen the rectal wall up to 16 cm from the anal verge (32); however, it may not be able to detect more cranial or proximal bowel lesions. On the other hand, MRI offers superior soft tissue contrast and is particularly valuable for visualizing deeper lesions that are located further cranially in the rectum and proximal bowel (33).

In comparison with previous meta-analyses, our study offers several important advantages. One of the most notable comparisons is with the work of Guerriero et al. (30), who included six studies that focused on comparing the performance of TVS and MRI in diagnosing DIE. Their findings, which indicated similar sensitivity and specificity between TVS and MRI, align with our meta-analysis results. However, the limited number of studies included in their analysis restricted the robustness of their conclusions. Moreover, their analysis involved comparisons across multiple anatomical sites, while we only focus on rectosigmoid site. In clinical practice, patients may present with lesions in multiple different sites. The preferred method for detecting multiple lesions may depend on clinical context, as each modality has its unique advantages.

Similarly, when comparing our study with Gerges et al. (29), there are clear strengths in our approach. Gerges et al. focused on comparing a variety of diagnostic tools, including TVS and MRI, but they performed an indirect comparison, which lowered the level of evidence and potentially introduced bias (29). In contrast, our meta-analysis focused exclusively on head-to-head studies, which strengthens the reliability of our conclusions. These improvements in methodology and study selection make our meta-analysis more reliable evidence for assessing the diagnostic performance of TVS and MRI in rectosigmoid DIE.

Both modalities have their own advantages and disadvantages. When considering the similar diagnostic performance, TVS appears to be a more cost-effective choice, given its higher availability and lower cost compared to MRI. TVS is non-invasive, widely accessible, and generally well-tolerated by patients, making it a preferred initial diagnostic tool in many settings (34). In addition to these advantages, one of the key benefits of TVS is its speed of detection. The ability to provide real-time imaging allows clinicians to quickly assess and identify lesions, which can be crucial in urgent clinical situations (35). In contrast, MRI, while offering superior soft tissue contrast and being highly effective in detecting deep-seated lesions, is more expensive, less accessible, and requires specialized equipment and expertise (36). However, the TVS is also limited by the diagnostic performance that depends on the operator’s experience. Regarding safety, both modalities are generally safe; however, MRI involves the use of strong magnetic fields, which may pose a risk for patients with certain implants, such as pacemakers (37), while TVS has minimal risk aside from the discomfort associated with the procedure. The complementary strengths of TVS and MRI should be considered, as each modality offers unique advantages in specific clinical scenarios. Despite these advantages and disadvantages, it should be noted that the high heterogeneity in the studies included in our meta-analysis, suggests that further researches focus in more specific patients are needed. It is important to note that while we compared the diagnostic performance of these two tools, each imaging technique has its own characteristics and strengths, leading to distinct diagnostic criteria. These differences are inherent and unavoidable. Furthermore, the adoption of a standardized classification system, such as #Enzian, in the evaluation of rectal endometriosis could facilitate more consistent reporting across studies (38). Its use would enhance comparability between sonographers and radiologists, ultimately improving the reliability and generalizability of diagnostic outcomes.

Beside rectosigmoid DIE, both TVS and MRI can effectively diagnose adenomyosis, with each modality presenting distinct diagnostic advantages, and with similar diagnostic performance (39). TVS offers real-time imaging and dynamic assessment capabilities, enabling clinicians to quickly evaluate uterine structures with immediate feedback. In contrast, MRI provides superior soft tissue contrast and comprehensive three-dimensional anatomical information, particularly useful for assessing the depth and extent of junctional zone alterations. However, on clinical grounds, the use of TVS would imply lower costs, faster examination times, and broader clinical accessibility, making it a preferred first-line imaging technique for initial adenomyosis screening (40). In addition, for rectosigmoid DIE, the commonly used intrasurgical laparoscopic ultrasound (IOUS) has demonstrated excellent diagnostic performance. It can be particularly advantageous during laparoscopic surgeries for real-time imaging, allowing for better visualization of endometriotic lesions and facilitating their identification and management (41). Future evaluations comparing IOUS with MRI and TVS may also be a worthwhile direction for discussion.

Several limitations of this meta-analysis should be acknowledged when interpreting the results. First, the heterogeneity observed across the included studies could have influenced the overall sensitivities and specificities of TVS and MRI. To address this, we performed meta-regression and leave-one-out sensitivity analysis to explore potential sources of heterogeneity, and our findings suggest that the region in which the studies were conducted may be a contributing factor. However, differences in healthcare infrastructure and patient situation from virous regions could have affected the diagnostic performance of both modalities. Second, approximately half of the included studies were retrospective design, which introduces the possibility of selection bias. Considering the retrospective nature of these studies, some data may not be homogeneous, leading to potential inaccuracies in the diagnoses. This limitation highlights the need for caution when interpreting the results, as the variability in data quality and collection methods across studies could impact the reliability of our findings. In terms of clinical practice, the results of this meta-analysis underscore the importance of both TVS and MRI as valuable diagnostic tools for rectosigmoid DIE. Given the similar diagnostic performance of these modalities, clinicians can consider TVS as a first-line imaging option, especially in settings where MRI may not be readily accessible due to cost or availability. Additionally, our findings suggest that future research should focus on developing standardized protocols that integrate both imaging techniques to enhance diagnostic accuracy and improve patient outcomes.

## 5 Conclusion

The meta-analysis reveals nearly equivalent diagnostic performance of TVS and MRI in detecting rectosigmoid DIE, with no statistical differences in sensitivity and specificity. However, high heterogeneity among studies highlights the need for further prospective research. Optimal diagnostic strategy for DIE requires comprehensive evaluation of imaging modalities’ distinctive characteristics. Clinicians must critically analyze the nuanced strengths and inherent limitations of each diagnostic technique to ensure patient-centered imaging selection.

## Data Availability

The original contributions presented in the study are included in the article/Supplementary material, further inquiries can be directed to the corresponding authors.
