# Supplementary material for: Transvaginal ultrasound and magnetic resonance imaging in detecting rectosigmoid deep infiltrating endometriosis: a comparative meta-analysis
Source: Front Med (Lausanne). 2025 Mar 17;12:1552185. doi: 10.3389/fmed.2025.1552185 (PMC11955469; doi:10.3389/fmed.2025.1552185)

Supplementary Table 1 Search strategy in PubMed and Embase.

| Database | Search strategy |
| --- | --- |
| PubMed | ("Endometriosis"[Mesh] OR "endometriosis"[Title/Abstract] OR “Endometrioma”[Title/Abstract] OR “Endometrioses”[Title/Abstract]) AND ("Ultrasonography"[Mesh] OR "ultrasonography"[Title/Abstract] OR "sonography"[Title/Abstract] OR “transvaginal ultrasound"[Title/Abstract] OR “TVS”[Title/Abstract]) AND ("magnetic resonance imaging"[Mesh] OR “MRI”[Title/Abstract] OR "magnetic resonance imaging"[Title/Abstract]) |
| Embase | ('endometriosis'/exp OR ‘endometriosis’:ab,ti) AND ('echography'/exp OR 'transvaginal echography'/exp OR ‘ultrasonography’:ab,ti OR ‘sonography’:ab,ti OR ‘transvaginal ultrasound’:ab,ti OR ‘TVS’:ab,ti) AND ('MRI scanner'/exp OR ‘MRI’:ab,ti OR ‘magnetic resonance imaging’:ab,ti) |

Supplementary Figure 1 Sensitivity analysis of TVS sensitivity using the leave-one-out method


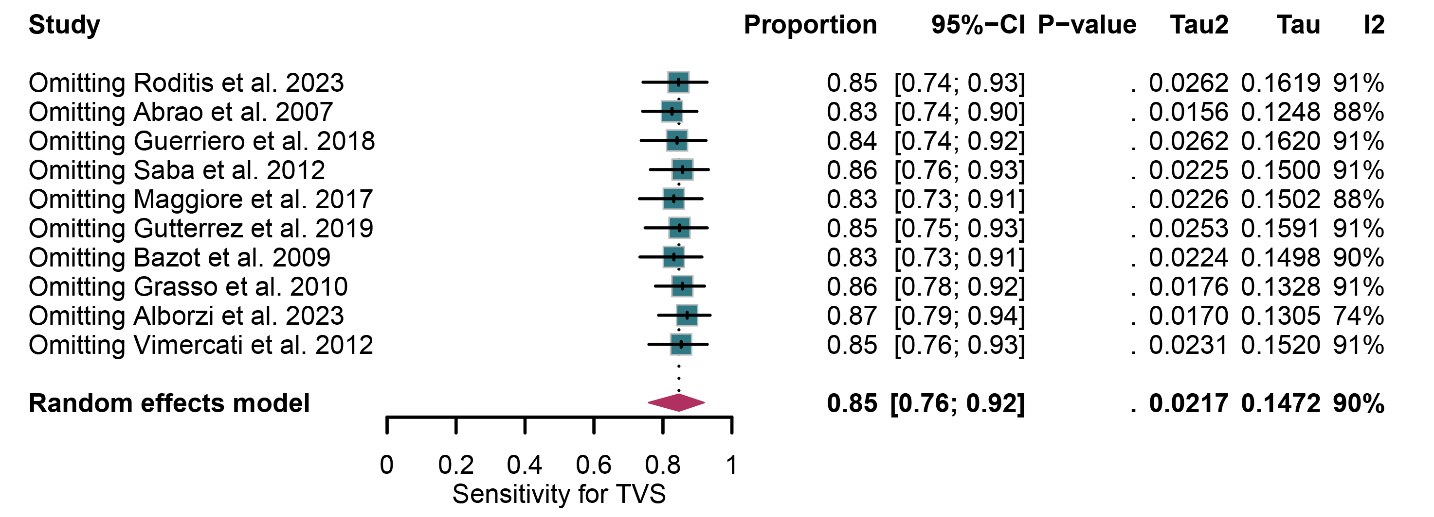


Supplementary Figure 2 Sensitivity analysis of MRI sensitivity using the leave-one-out method


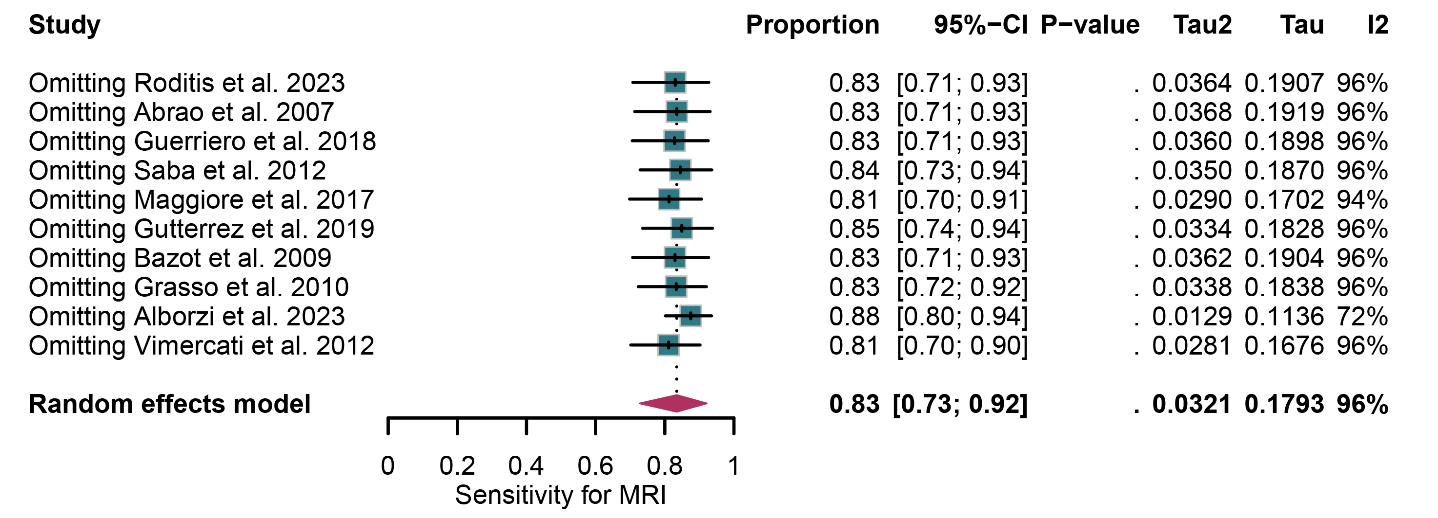


Supplementary Figure 3 Sensitivity analysis of TVS specificity using the leave-one-out method


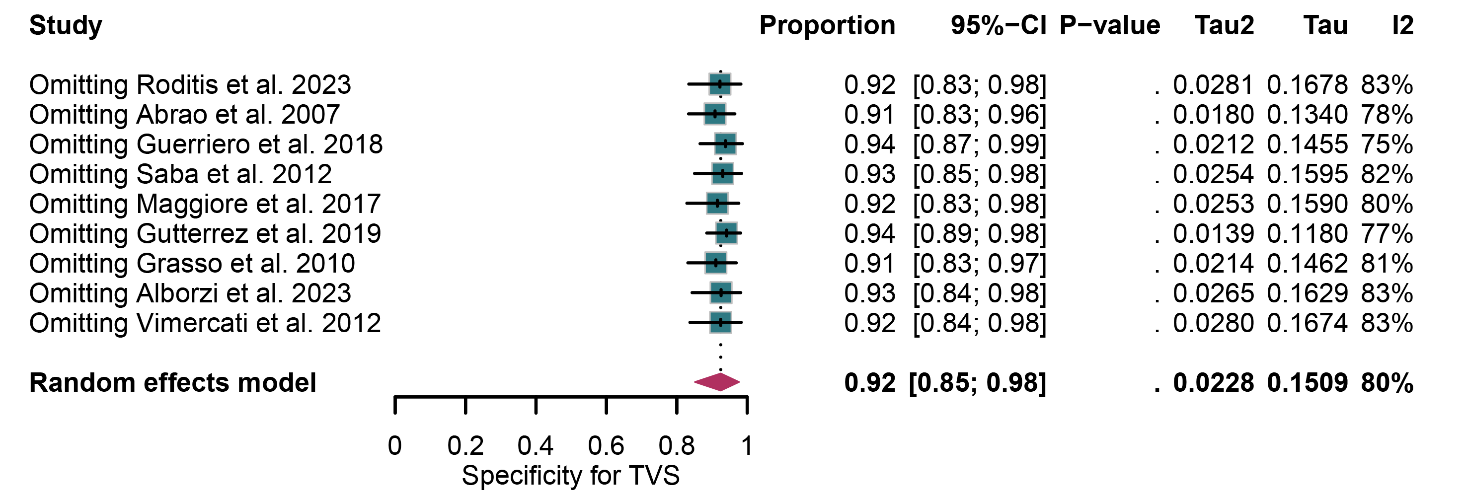


Supplementary Figure 4 Sensitivity analysis of MRI specificity using the leave-one-out method


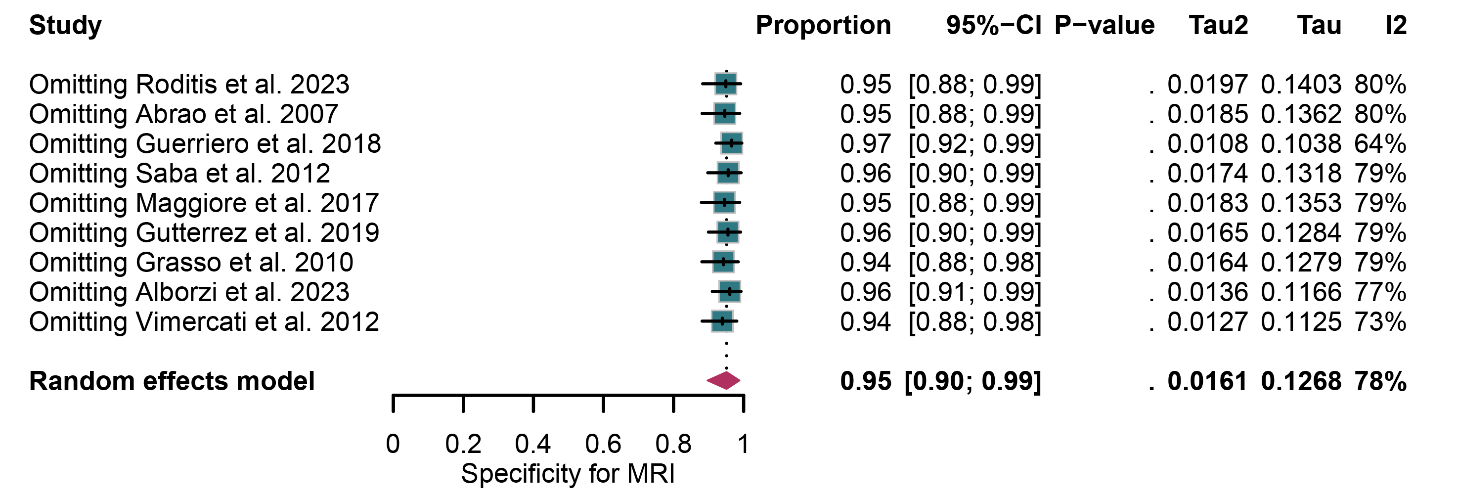


Supplementary Figure 5 Funnel plot of TVS sensitivity


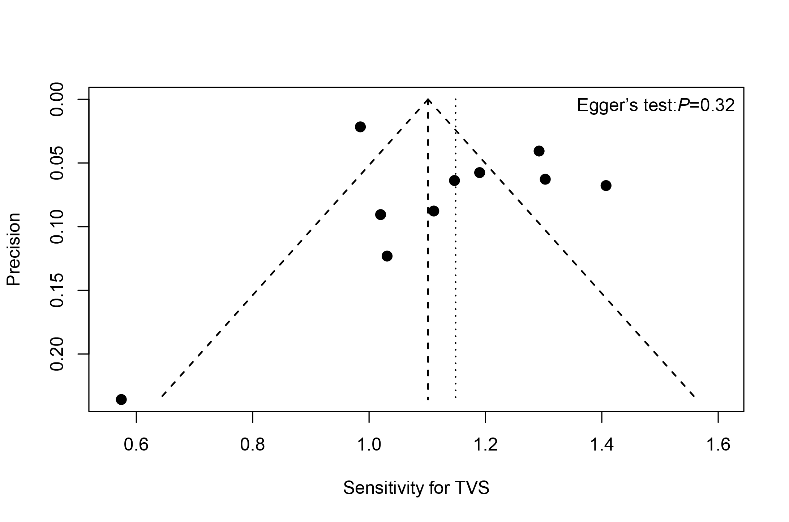


Supplementary Figure 6 Funnel plot of MRI sensitivity


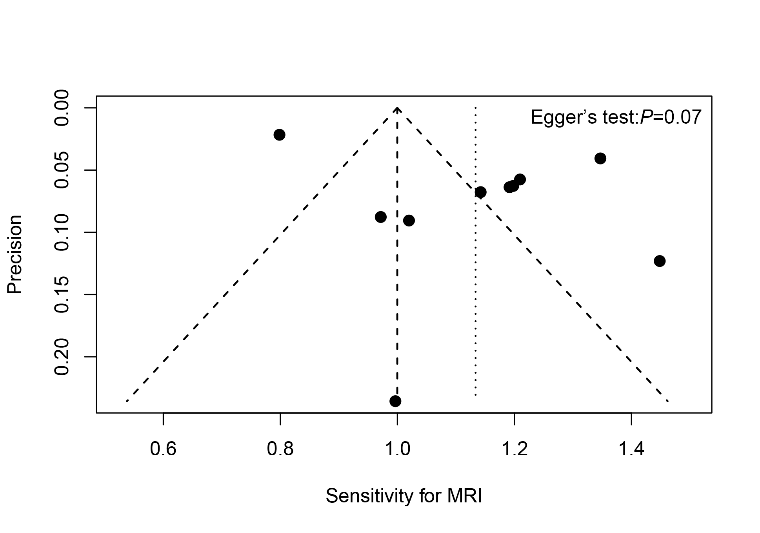


Supplementary Figure 7 Funnel plot of TVS specificity


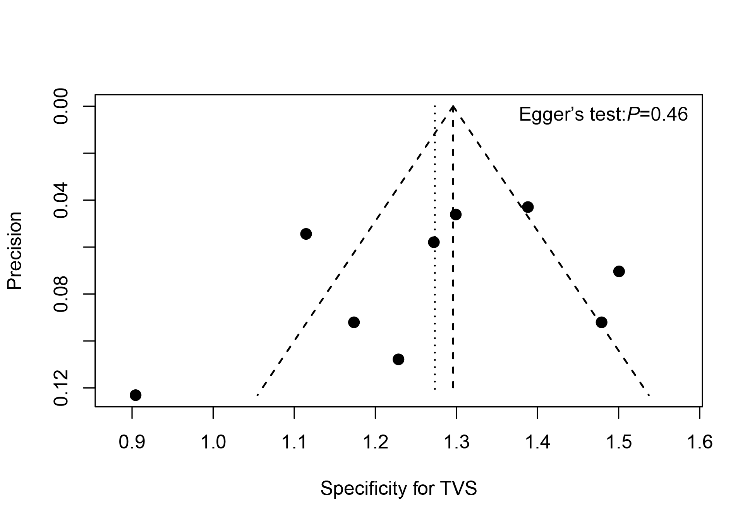


Supplementary Figure 8 Funnel plot of MRI specificity


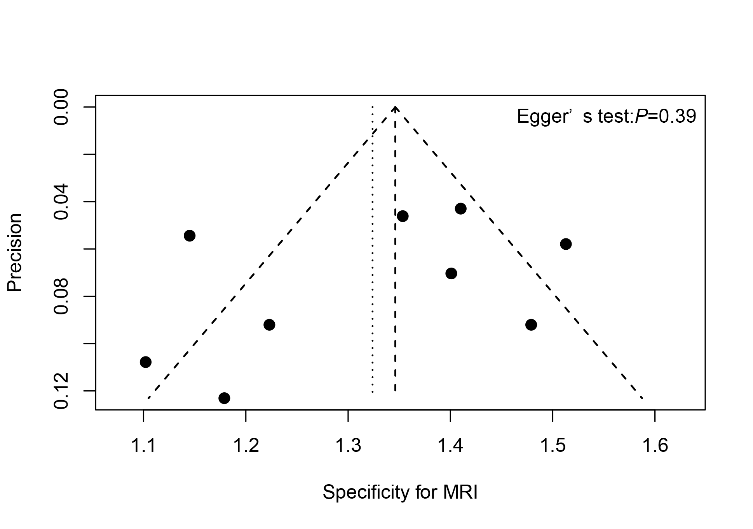

Supplement: Supplementary file 1 [file Data_Sheet_1.docx]
